# Anaphylaxis in elderly people

**DOI:** 10.1097/ACI.0000000000000855

**Published:** 2022-09-14

**Authors:** Maria Teresa Ventura, Elisa Boni, Luis Taborda-Barata, Hubert Blain, Jean Bousquet

**Affiliations:** a Unit of Geriatric Immunoallergology, University of Bari Medical School, Bari, Italy; b Allergologia e Autoimmunità LUM, Maggiore Hospital, AUSL Bologna, Italy; c UBIAir – Clinical & Experimental Lung Centre, University of Beira Interior, Covilhã and CICS-Health Sciences Research Centre, University of Beira Interior, Covilhã, Portugal; d Department of Immunoallergology, Cova da Beira University Hospital Centre, Covilhã, Portugal; e Department of Geriatrics, Montpellier University Hospital, MUSE, Montpellier, France; f Institute of Allergology, Charité – Universitätsmedizin Berlin, Corporate Member of Freie Universität Berlin and Humboldt-Universität zu Berlin, Berlin, Germany; g Fraunhofer Institute for Translational Medicine and Pharmacology ITMP, Allergology and Immunology, Berlin, Germany; h University Hospital Montpellier, France

**Keywords:** adrenaline, Anaphylaxis, elderly, older

## Abstract

**Purpose of review:**

Anaphylaxis is common in old-age adults but is insufficiently understood by physicians, and may be underdiagnosed. This review discusses the specificities of anaphylaxis in this age group and stresses the importance of adrenaline in its management.

**Recent findings:**

Data from the European Anaphylaxis Registry on elderly patients is a major finding. Other findings include the prevention of possible anaphylactic reactions in coronavirus disease 2019 vaccination as well as some new epidemiologic data.

**Summary:**

The most common risk factors are hymenoptera venom and food and drug allergy. Cardiovascular symptoms are the most important ones to reverse in old-age adults, especially due to the multiple comorbidities. Anaphylaxis in old-age adults has a more severe outcome than in younger ones. Polypharmacy is a specific factor to be considered. The Airway, Breathing, Circulation, Disability, Exposure (ABCDE) algorithm is applicable in all clinical emergencies for immediate assessment and treatment, and should be considered for all patients. Adrenaline is the mainstay of the management of the condition. There are no absolute contraindications to the prescription of self-injectable adrenaline in elderly individuals at risk of anaphylaxis.

## INTRODUCTION

Anaphylaxis is a life-threatening condition which can affect people at any age. It is the most severe form of allergy and is usually mediated by an immunoglobulin E (IgE) immune response. As life expectancy and the number of older adults become higher, allergic diseases show an increasing incidence among the geriatric population [1].

Clinical presentation, prognosis and eliciting factors may differ according to age. In this review, we attempt to focus on these issues.

The immunological system is influenced by aging, as are other organs and systems. It seems that these changes may be responsible for anaphylaxis and allergic adverse drug reactions in older adults [2]. Age over 65 is a well established factor that increases the risk for near-fatal or fatal anaphylaxis [3]. Indeed, anaphylaxis is more life-threatening in this age group, and more intensive medical care is often required [4^▪▪^,^5^].

This paper follows a recent review on the management of anaphylaxis following coronavirus disease 2019 (COVID-19) vaccination in older adults [6^▪▪^]. 

**Box 1:**
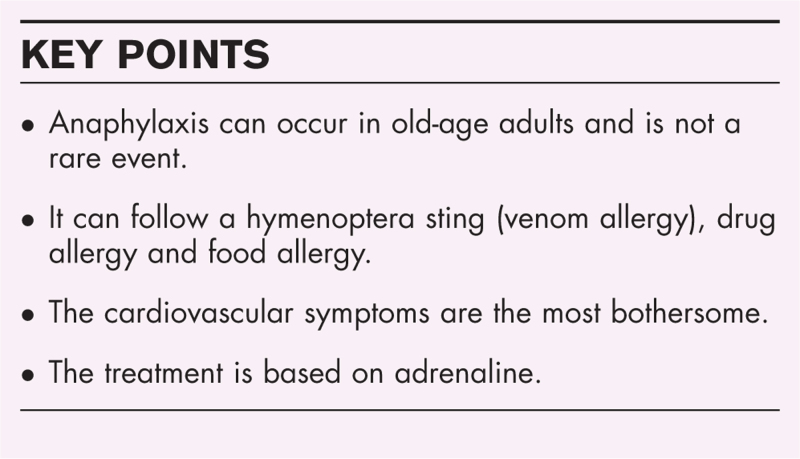
no caption available

## EPIDEMIOLOGICAL DATA

In an epidemiological study conducted in the United Kingdom on anaphylaxis elicited by drugs over a 10-year period, it was observed that the incidence of anaphylaxis was higher in individuals aged 60 or older, and that the risk of fatal outcome was greater in this age group. Drug-related causes (particularly antibiotics) were the most common causes of hospital admission and fatality in the elderly population [7].

Arroyo *et al.*[8^▪^] performed a cross-sectional analysis of trends in the US emergency departments among older adults between 2006 and 2014. It was observed that anaphylaxis-related visits and hospitalizations, especially drug-related, have increased over time. The author found that older adults had a higher anaphylaxis-related hospitalization rate compared with that of younger adults. Risk factors for fatal anaphylaxis were older age, especially 85 years of age, and drug exposure.

Between 2007 and 2017, the European Anaphylaxis Registry collected data from 6,891 cases, of which 1,123 (16.3%) were older than 65 years. These data were provided by tertiary referral centers specialized in allergology in eight European countries [4^▪▪^,^9^,^10^]. The median age was 70 years, and 95% of the cases were between 65 and 80 years. This age group included a higher percentage of male individuals in comparison with the adult group. Moreover, elderly people more frequently suffered from cardiovascular, thyroid and malignant diseases. In contrast, atopic diseases were significantly less common than in the younger adult group.

## ELICITING FACTORS

### Hymenoptera venom allergy

In Europe, hymenoptera venom allergy is the first cause of anaphylaxis in older adults, with *Vespula* spp. venom being the cause of the majority of cases [4^▪▪^]. Older age is a well established risk factor for severe systemic reactions to wasp and bee stings [11,12]. Sturm *et al.*[13] performed an open, prospective, observational multicenter study aimed at evaluating – among other objectives – whether antihypertensive drugs represent a risk factor for severe sting reactions and systemic adverse events to venom immunotherapy. Of all included patients, 388 took antihypertensive drugs: 10.4% ß-blockers, 11.9% angiotensin converting enzyme inhibitors (ACEI), 5% ACEI and ß-blockers. The authors showed that the prevalence of cardiovascular diseases or hypertension was not a risk factor for systemic adverse events to venom immunotherapy [13]. Concomitant treatment with ß-blockers or ACEI had no influence on the severity of sting reaction in patients not yet under desensitization treatment. In addition, such antihypertensive drugs were not associated with increased risk of adverse events to immunotherapy [14].

In the study by Sturm *et al.*[13], the presence of cardiovascular diseases or hypertension appeared to be a risk factor for systemic reactions to insect sting. However, when age was taken into account, the association between cardiovascular disease and severity of systemic reaction to sting became nonsignificant.

### Drug allergy

Older individuals have an increased risk for developing drug adverse reactions. However, studies on the epidemiology of drug allergy in older adults are scarce. Moreover, a bias of many studies is represented by an incorrect definition of drug hypersensitivity reactions, since most of the described adverse drug reactions are not allergic. The evidence suggests that drug allergy has a prevalence of 0.6–2.1% in older adults but constitutes 10% of drug-related deaths [15].

In European countries, drugs are the second cause of anaphylaxis among adults, and are more prevalent in older adults. Analgesics (metamizole, diclofenac, ibuprofen) and antibiotics (penicillin, cephalosporins, quinolones) are the most frequent drugs associated with anaphylaxis in this age group [4^▪▪^]. On the other hand, drug-related anaphylaxis appears to be the first cause of Emergency Department visits in the United States [8^▪^,16^▪^].

Rapid drug desensitization can be used in old-age adults to address hypersensitivity reactions to chemotherapeutics and monoclonal antibodies, allowing patients to be treated with optimal pharmacological agents [17,18].

### Food allergy

Food is considered the least common cause of anaphylaxis in older adults [19]. In fact, according to the European Anaphylaxis Registry, when the geriatric age group was considered, only 11% of anaphylactic reactions were caused by food, whereas this cause accounted for 22% of the cases in younger adults. The main culprit allergens are represented by wheat (14%) and hazelnut (13%) [4^▪▪^].

### Reactions to the coronavirus disease 2019 vaccine

The safety of the COVID-19 vaccine in older adults was tested in clinical trials [20^▪^]. Allergic or anaphylactic reactions are a rare event and it does not appear that these reactions are more common in older adults.

## CLINICAL PRESENTATION

In the European Anaphylaxis Registry, anaphylactic symptoms were similar in younger and older adults. However, cardiovascular symptoms occurred more frequently in older adults (80% compared to 75% in younger adults). This finding is in line with other studies [19,21]. A major cardiovascular symptom was loss of consciousness (33%). In this registry, just like in younger individuals, skin symptoms were the most common clinical manifestation. Urticaria and angioedema are two symptoms of anaphylaxis and usually appear before any other symptoms. However, in the registry, skin symptoms were significantly less frequent in older than in younger adults. Furthermore, the severity of anaphylactic reactions in older patients without skin symptoms was increased in comparison with younger adults. Cyanosis, syncope, and dizziness are highly predictive of shock development in older people. In the registry, severe anaphylactic reactions, including grade III (47%) and grade IV (4%) of the anaphylaxis Ring and Messmer classification [22], were more prevalent in older people. Anaphylaxis in the registry was graded according to Ring and Messmer [22], although there are proposals for new grading systems [23,24], and WHO and regulatory authorities recommend the use of the Brighton Collaboration Anaphylaxis Working Group for pharmacovigilance registers [25].

**FIGURE 1:**
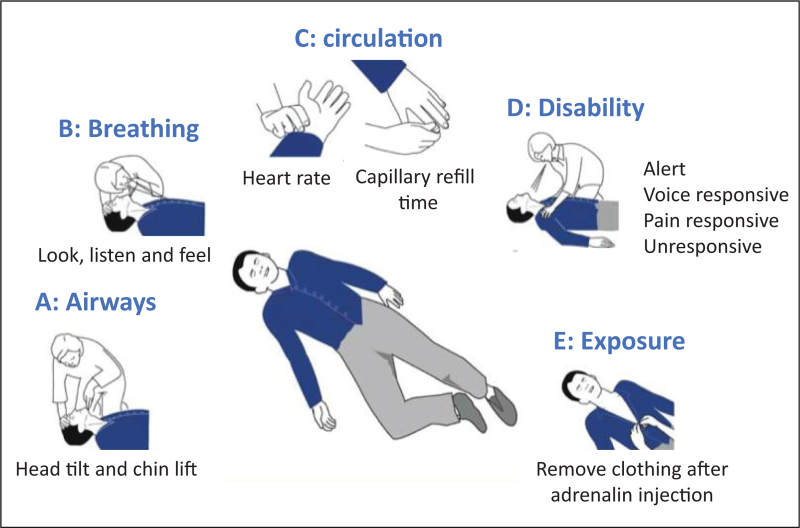
Airway, Breathing, Circulation, Disability, Exposure (ABCDE) algorithm. From ref. [6^▪▪^].

Significantly more older people as compared with younger and middle-aged adults with grade II and III anaphylaxis needed hospitalization and ICU care [22] (Fig. 1)

## RISK FACTORS FOR SEVERITY OF ANAPHYLAXIS IN OLDER PEOPLE

Several factors in the geriatric population contribute to increased risk for adverse drug reactions: female sex, chronic diseases, frailty, memory issues, and treatment with multiple medications [26]. Considering the different triggers of anaphylaxis (excluding the confounding factor of concomitant cardiovascular or other diseases), higher age has been consistently associated with increased rates of fatal drug anaphylaxis. This may be related to an increased prevalence of drug allergy following an increased drug exposure, and/or to an increased underlying cardiovascular vulnerability [27].

### Comorbidity

In the European Anaphylaxis Registry, concomitant mastocytosis [28] was the most important predictor for an increased risk of severe anaphylaxis [4^▪▪^,^9^,^10^,^29^].

The onset of cardiovascular involvement and the occurrence of anaphylaxis-related deaths can be attributed to a greater susceptibility to mast cell-derived mediators on the cardiovascular system [30]. Anaphylaxis is more severe and represents an increased death risk in patients with coronary artery disease because the number of mast cells and the production of their vasoactive mediators are increased in ischemic cardiomyopathy. Mast cell released mediators can (i) induce modification of the arterial system through vasospasm of large coronary arteries, (ii) influence the vasomotor tone of small intramural coronary arteries, and (iii) lead – via direct dysrhythmogenic effects – to a global reduction of myocardial blood flow [31]. In addition, atherosclerotic lesions make coronary arteries more susceptible to the effects of mast cell- and basophil-derived mediators [32]. Furthermore, individuals with an underlying vascular illness are less tolerant to hypoxia and hypotension during anaphylaxis.

Hereditary alpha-tryptasemia, an autosomal dominant trait, existing in around 5–8% of the population, is another risk factor for more severe anaphylaxis. This concerns not only patients with systemic mastocytosis, but also those with venom immunotherapy-related and idiopathic anaphylaxis [33].

In the European Anaphylaxis Registry, cardiovascular diseases, thyroid diseases and cancer were more common in older than in younger adults [10].

### Polypharmacy and medications used in older people

Older adults are more likely to take many different drugs, by oral route and particularly by injection, than younger ones. Brown *et al.*[34] showed that, in a large cohort of people admitted to Emergency Departments, the intake of medication and older age were significantly correlated with hypotensive reactions. Antihypertensive drug intake correlates with higher organ system involvement during anaphylaxis and greater risk of hospital admission, regardless of age [21].

In recent years, solid evidence has been provided in the field of insect venom allergy that the use of ß-blockers or ACEI is not associated with increased severity of sting reactions in patients with venom allergy [13]. However, in the European Anaphylaxis Registry, medications associated with an increased risk of severe anaphylaxis “risk cofactors” – such as ACEI, AT-2-antagonists (angiotensin II receptor type 2), ß-blockers, acetylcholine, and proton pump inhibitors – were significantly more frequently prescribed in older people (57%) than in younger adults (18%) [9]. Independently of the age of the patient, ß-blockers and ACEI administered close to allergen immunotherapy increased the risk of developing severe anaphylaxis, while aspirin and AT-2 did not [9]. However, a systematic review with low quality evidence showed that ß-blockers and ACEI increased the severity of anaphylaxis, due to differences in confounders, particularly cardiovascular diseases [35].

It is important to stress that many older adults are treated with anxiolytics, antidepressants, or hypnotics that may alter the individual's recognition and perception of anaphylaxis symptoms [36].

## MANAGEMENT OF ANAPHYLAXIS IN OLDER PEOPLE

### The Airway, Breathing, Circulation, Disability, Exposure approach

The Airway, Breathing, Circulation, Disability, Exposure (ABCDE) algorithm is applicable in all clinical emergencies for immediate assessment and treatment [37] (Fig. 1 from [6^▪▪^]). If anaphylaxis is suspected, every patient should receive a rapid evaluation of vital functions via ABCDE, and problems should be addressed in a targeted manner.

The aims of the ABCDE approach are [37]:

- to provide life-saving treatment,
- to break down complex clinical situations into more manageable parts,
- to serve as an assessment and treatment algorithm,
- to establish common situational awareness among all treatment providers,
- to buy time to establish a final diagnosis and treatment.

### Adrenaline in older people

Guidelines from EAACI [38] and the World Allergy Organization [39] recommend a prompt intramuscular injection of adrenaline as first-line therapy for anaphylaxis at any age. Adrenaline is able to counteract most of the severe anaphylactic symptoms in older adults [40]. Intramuscular administration of adrenaline, if possible using a ready-to-use preparation or auto-injector, is recommended [6^▪▪^]. After an initial dose of 0.3–0.5 ml of a 1:1000 dilution (1 mg/ml), the patient should then be monitored, and, if ineffective, the administration should be repeated after at least 5 min [40]. The subcutaneous route should not be used because the vasoconstrictor effect of adrenaline injected into the subcutaneous tissue may delay adrenaline absorption [41]. The intra-vascular route should be avoided since most adrenaline cardiovascular side events appear to occur via this route [42]. Intravenous continuous infusion should only be given to patients not responding to intramuscular injection, and under careful ECG monitoring [38].

In older adults with cardiovascular disease, the benefits versus the harms of adrenaline injection should be weighed swiftly and carefully in order to provide the treatment without delay. However, cardiovascular diseases do not exclude the use of adrenaline since no other medications have life-saving effects [32]. There are no absolute contraindications to the prescription of self-injectable adrenaline in older adults or in those with a cardiovascular disease who are at risk of anaphylaxis. Serious adverse effects, such as ventricular arrhythmias, hypertension or myocardial ischemia, have not been reported following the use of adrenaline autoinjectors [43]. However, in older adults with cardiovascular disease, a cardiologic consultation might be indicated. Limited mobility or the presence of joint diseases could reduce the ability to use the auto-injector, and this issue should be considered when prescribing this first-line treatment. Older adults with anaphylaxis seem to be more prone to experiencing a cardiac adverse event after adrenaline injection, particularly those older than 80 years who have the highest risk [44].

In the European Anaphylaxis Registry, adrenaline was administered in 30% of older patients. Hospitalization was required in 60%, and 19% were treated in an ICU. Moreover, the prescription of self-injectable adrenaline was lower in older adults.

### Other treatments

Regular intake of multiple medications is frequent in older patients (polypharmacy). Co-medication may modify the evolution of anaphylaxis, as well as its management. The therapeutic effect of adrenaline may be blunted by ß-blockers. In this situation, if adrenaline is not effective, glucagon can be administered intravenously, as it has a mechanism of action independent of the ß-receptors [45,46].

An ARIA-EAACI-EuGMS (Allergic Rhinitis and its Impact on Asthma, European Academy of Allergy and Clinical Immunology, European Geriatric Medicine Society) working group has proposed some recommendations for older adults receiving the COVID-19 vaccine [6^▪▪^].

## CONCLUSION

Allergic diseases are usually thought to be specific in young individuals. However, it is common that allergies often persist into older age, and occasionally their actual onset can occur in old age.

It seems clear that age over 65 is a factor that increases the risk for severe, near-fatal or fatal anaphylaxis [3]. Older age also correlates with different clinical presentation, prognosis and eliciting factors in comparison with the younger adult population.

Anaphylaxis in the geriatric population is often related to insect venoms and drugs. Clinical features of anaphylaxis in these patients often include cardiovascular symptoms and adrenaline administration. In addition, hospitalization is frequently required [4^▪▪^,8^▪^].

Moreover, genetic factors, hormonal imbalances, nutritional deficiencies or inflammation may affect the immune system, inducing changes in immunological responses. Diagnosis and management of allergic diseases in older adults can also differ due to several factors such as comorbidities, multiple concomitant medications and frailty [40]. Whether concomitant medications such as β-blockers or ACEI are able to aggravate anaphylaxis is still being debated [3]. Finally, recent studies confirm that older age and drug-related triggers are all consistently associated with severity of anaphylaxis and death [8^▪^,16^▪^].

However, in order to identify targeted interventions to improve outcomes in this vulnerable population, larger studies are necessary to investigate anaphylaxis elicitors and risk factors.

It should be borne in mind that the intramuscular injection of adrenaline, frequently underused, is the main treatment for anaphylaxis, at any age. There are no absolute contraindications to the prescription of self-injectable adrenaline in older adults at risk of anaphylaxis. However, adrenaline is still frequently underused in older individuals, and the reasons underlying this problem should be further addressed and corrected.

## Acknowledgements

*None.*

### Financial support and sponsorship

*None.*

### Conflicts of interest

*There are no conflicts of interest.*

## REFERENCES AND RECOMMENDED READING

Papers of particular interest, published within the annual period of review, have been highlighted as:

▪ of special interest
▪▪ of outstanding interest
